# Effect of histamine-2-receptor antagonists versus sucralfate on stress ulcer prophylaxis in mechanically ventilated patients: a meta-analysis of 10 randomized controlled trials

**DOI:** 10.1186/cc9312

**Published:** 2010-10-29

**Authors:** Jiahao Huang, Yunfei Cao, Cun Liao, Liucheng Wu, Feng Gao

**Affiliations:** 1 Department of Colorectal and Anal Surgery, First Affiliated Hospital, Guangxi Medical University, 22 Shuangyong Road, Nanning 530021, Guangxi, PR China

## Abstract

**Introduction:**

We conducted a meta-analysis in order to investigate the effect of histamine-2-receptor antagonists (H_2_RA) versus sucralfate on stress ulcer prophylaxis in mechanically ventilated patients in the intensive care unit (ICU).

**Methods:**

A systematic literature search of Medline, EMBASE, Cochrane Central Register of Controlled Trials (1966 to January 2010) was conducted using specific search terms. A review of Web of Science and a manual review of references were also performed. Eligible studies were randomized control trials (RCTs) that compared H_2_RA and sucralfate for the prevention of stress ulcer in mechanically ventilated patients. Main outcome measures were rates of overt bleeding, clinically important gastrointestinal (GI) bleeding, ventilator-associated pneumonia, gastric colonization and ICU mortality.

**Results:**

Ten RCTs with 2,092 participants on mechanical ventilation were identified. Meta-analysis showed there was a trend toward decreased overt bleeding when H_2_RA was compared with sucralfate (OR = 0.87, 95% CI: 0.49 to 1.53). A total of 12 clinically important GI bleeding events occurred among 667 patients (1.8%) in the H_2_RA group compared with 26 events among 673 patients (3.9%) in the sucralfate groups. Prophylaxis with sucralfate decreased the incidence of gastric colonization (OR = 2.03, 95% CI: 1.29 to 3.19) and ventilator-associated pneumonia (OR = 1.32, 95% CI: 1.07 to 1.64). Subgroup analysis showed H_2_RA was not superior to sucralfate in reducing early-onset pneumonia (OR = 0.62, 95%CI: 0.36 to 1.07) but had a higher late-onset pneumonia rate (OR = 4.36, 95%CI: 2.09 to 9.09) relative to sucralfate. No statistically significant reduction was observed in mortality of ICU between groups (OR = 1.08, 95% CI: 0.86 to 1.34).

**Conclusions:**

In patients with mechanical ventilation, H_2_RA resulted in no differential effectiveness in treating overt bleeding, but had higher rates of gastric colonization and ventilator-associated pneumonia. Additional RCTs of stress ulcer prophylaxis with H_2_RA and sucralfate are needed to establish the net benefit and risks of adverse effect in mechanically ventilated patients.

## Introduction

Stress-related mucosal damage might develop in the stomach and duodenum and progress to ulceration within 4 to 5 days after injury. Intensive care unit (ICU) patients are prone to develop stress-related gastrointestinal (GI) hemorrhage, which is associated with increased morbidity and mortality. Respiratory failure, hypotension, and coagulopathy are the strongest risk factors for clinically important GI bleeding [1-4], especially for those patients with prolonged mechanical ventilation, who have a 4- to 21-fold risk of stress ulceration compared with those patients without prolonged mechanical ventilation [5,6]. Therefore, prophylaxis against stress ulceration traditionally has been recommended for the prevention of upper GI hemorrhage in critically ill patients. Antacids, which are the first agents employed to significantly decrease the incidence of stress ulcer, have been widely displaced by histamine-2-receptor antagonists (H_2_RA) and sucralfate because of the excessive nursing-time demand that results from frequent dosing and gastric pH testing. H_2_RA, such as ranitidine and cimetidine, blocks the secretion of gastric acid and raises the gastric pH, promoting the proliferation of bacteria - particularly, Gram-negative bacilli, tracheobronchial colonization, and nosocomial pneumonia - in the stomach [7-9]. Sucralfate, which does not alter gastric pH, exerts its topical effect on ulcer disease by binding to the proteins of the ulcer site. Cook and colleagues [10] found, in contrast, that universal prophylaxis may not be warranted as only 1 patient out of 1,000 treated would benefit from the prophylaxis. Saint and Matthay [11] considered that stress ulceration prophylaxis never demonstrated a benefit in decreasing the incidence of mortality.

To our knowledge, no previous systematic review in stress ulcer prophylaxis has definitively established whether H_2_RA and sucralfate decrease clinically important GI bleeding, nor has any study generated clinical recommendations of different prophylactic regimens. Cook and colleagues [12] conducted a meta-analysis of the effect of stress ulcer prophylaxis and identified a trend toward a decreased incidence of nosocomial pneumonia when sucralfate was compared with H_2_RA. Moreover, a recent meta-analysis [13] reported a significantly increased risk of pneumonia with ranitidine compared with sucralfate. The study, however, was limited by small sample size (in particular, of patients with pneumonia) and therefore might not be reliable. In addition, the end point of 'clinically important GI bleeding' was not homogeneous in the trails included in the study [12]. To reconcile the inconsistencies in the prior studies, we attempted to summarize the available randomized controlled trials (RCTs) and gain adequate sample size and power by combining the results of several studies in a rigorous scientific overview that compared H_2_RA and sucralfate. We attempted to ascertain the frequencies of overt bleeding, clinically important GI bleeding, occurrence of ventilator-associated pneumonia (VAP), gastric colonization, and ICU mortality in a large series of mechanically ventilated patients in the ICU.

## Materials and methods

### Data sources

A comprehensive search was performed to identify RCTs in Medline, Embase, the Cochrane Central Register of Controlled Trials (CENTRAL), and Web of Science in any language between 1966 and January 2010. The following search terms, alone or in combination, were used: stress ulcer, histamine-2-receptor antagonists, ranitidine, cimetidine, famotidine, sucralfate, mechanical ventilation, and randomized controlled trials. No language restrictions were imposed. An independent search using Web of Science was conducted to ensure that all relevant clinical trials were included in the meta-analysis. In addition, bibliographies of retrieved articles were manually searched for other relevant studies.

### Study selection

Clinical trials that met the following criteria were included in the meta-analysis: (a) randomized trials of an H_2_RA (including ranitidine, cimetidine, and famotidine) compared with sucralfate, (b) trials with adults who were projected to require mechanical ventilation for at least 48 hours in the ICU, and (c) trials with available data on the proportion of patients with overt bleeding, clinically important GI bleeding, and VAP or with gastric colonization and ICU mortality. Applying these prespecified inclusion criteria, two investigators independently reviewed all potentially relevant articles, and disagreement among investigators was resolved by consensus. When two studies had substantial overlap in terms of investigator, institution, and study population, the one that was more recent and of better quality was included.

### Quality assessment

Two reviewers independently evaluated each study while using a critical review checklist of the Dutch Cochrane Centre [14]. The following methodological features most relevant to the control of bias were assessed: adequate sequence generation, allocation concealment, blinding, selective outcome reporting, and other sources of bias. Each criterion was categorized as 'yes', 'no', or 'unclear', and the summary assessments of the risk of bias for each important outcome within and across studies were categorized as 'low risk of bias', 'unclear risk of bias', or 'high risk of bias'.

### Data extraction

Two independent reviewers abstracted the data in a traditionalized format. The following information was sought from each article: first author identification, year of publication, country, study duration, sample size, duration of patient follow-up, participant characteristics (patient number and mean age), Acute Physiology and Chronic Health Evaluation II (APACHE II) score (range of scores was 0 to 71, with higher scores indicating a more severe illness) [15], and intervention (drug and dose). Discrepancies in data extraction were to be resolved by consensus, referring back to the original article, and by contacting the study authors if necessary.

The primary end points of the meta-analysis were overt bleeding, clinically important GI bleeding, and VAP in the population of patients who received H_2_RA therapy in comparison with those who received sucralfate. Secondary end points were gastric colonization and ICU mortality.

In this study, overt bleeding was defined as signs of hematemesis, nasogastric aspirate containing blood or coffee-ground material, melena, or hematochezia, the last of which was a potential problem in the mechanically ventilated patients as a result of stress ulceration. Clinically important GI bleeding was defined as overt bleeding accompanied by at least one of the following: (a) a decrease in blood pressure of 20 mm Hg within 24 hours of bleeding, (b) a decrease in blood pressure of 10 mm Hg and an increase in heart rate of 20 beats per minute on orthostatic change after upper GI bleeding, or (c) a decrease in hemoglobin of 20 g/L and transfusion of 2 units of blood within 24 hours or gastric bleeding requiring surgery. We included the studies that precisely met the definition of 'ventilator-associated pneumonia' according to Cook and colleagues [16]. The early-onset and late-onset pneumonias were diagnosed if they occurred during the first 4 days before or 4 days after the initiation of mechanical ventilation, respectively. Consequently, only patients observed for more than 4 days could be evaluated for the development of late-onset pneumonia. A patient was considered to have gastric colonization with high counts when quantitative culture of at least one specimen had more than 100 colony-forming units/mL, and ICU mortality was considered to occur as death from any cause between the date of random assignment and the end of the active study phase in the ICU.

### Data synthesis

Version 9.2 of the Stata program (StataCorp LP, College Station, TX, USA) was used for statistical analysis. Data were analyzed by an intention-to-treat analysis, so all patients who were randomly allocated to one treatment arm or the other were analyzed together regardless of whether they completed, or indeed received, regimens. To standardize reporting of our results, odds ratios (ORs) and 95% confidence intervals (CIs) were calculated from raw data of every trial. For the meta-analysis, we initially used the fixed-effects model [17], based on inverse variance weights for combined results from the individual trials. The Cochran χ^2 ^and the I^2 ^statistic were first calculated to assess the heterogeneity among the proportions of the included trials. If the *P *value was less than 0.1 and I^2 ^was greater than 50%, the assumption of homogeneity was deemed invalid, and the following techniques were employed to explore the heterogeneity: (a) subgroup analysis, (b) sensitivity analysis performed by omitting one study in each turn and investigating the influence of a single study on the overall meta-analysis estimate when necessary, and (c) if the heterogeneity still existed, randomized-effects models as described by DerSimonian and Laird [18] were applied to incorporate between-study heterogeneity in addition to sampling variation for the calculation of summary OR estimates and corresponding 95% CIs. Otherwise, the pooled event rate data for each treatment group were presented alongside the common OR results obtained from the pooled analysis in the fixed-effects model. The Egger regression test, Begg adjusted rank correlation test, and visual inspection of a funnel plot were performed to assess publication bias [19,20]. A two-tailed *P *value of less than 0.05 was considered statistically significant. Circumstances that might bring about clinical heterogeneity included differences in severity of disease, intervention dosage, measurements, and management. This work was performed in accordance with the Quality of Reporting of Meta-analyses (QUOROM) guidelines for meta-analysis of randomized clinical trials [21].

## Results

### Study characteristics

**Figure 1:**
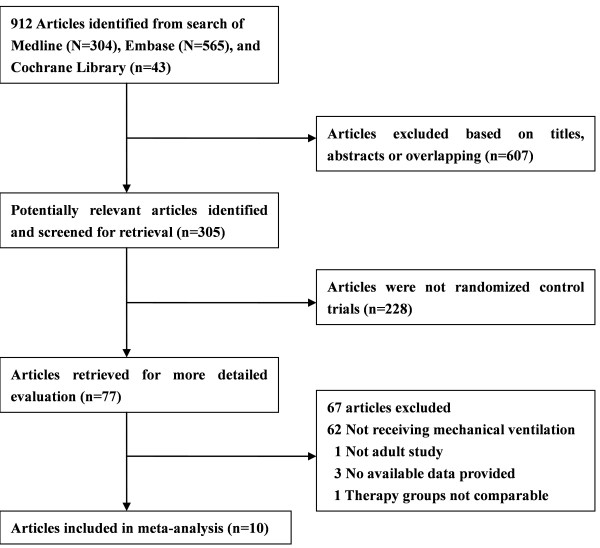
**Flowchart of study selection**.

The search strategy generated 912 references: Medline (*n *= 304), Embase (*n *= 565), and CENTRAL (*n *= 43). A total of 77 potentially eligible studies were identified by literature search. We excluded 62 studies in which participants did not receive mechanical ventilation in the ICU. Three studies were excluded because they failed to report adequate data, one was based on pediatric population, and one paper did not have comparable therapy groups. Finally, 10 remaining trials [16,22-30] were determined to have met the inclusion criteria and were invited to collaborate. The flowchart of the literature search of this meta-analysis is shown in Figure 1. Eight trials tested ranitidine therapy versus sucralfate, and one trial examined famotidine versus sucralfate. Of the 2,092 participants, 1,041 were randomly assigned to H_2_RA (970 received ranitidine and 71 received famotidine) and 1,051 were randomly assigned to sucralfate. Details of the included studies are summarized in Table 1. Patient enrollment ranged from 16 to 604, mean age of patients ranged from 26.8 to 60.0 years, and the duration of follow-up ranged from 7 to 27.3 days. Ranitidine doses ranged from 150 to 300 mg/day, and sucralfate doses ranged from 4 to 6 g/day. Participants included ICU patients, who required mechanical ventilation for more than 2 days. Patients' baseline characteristics in treatment groups were well balanced according to APACHE II score.

**Table 1:** Baseline characteristics of the trials

Study	Country	Study design	Duration	Follow-up	Treatment groups	APACHE II score	Patients			Intervention
								
							Number	Age, years	Males/Females	
Prakash et al. [22], 2008	India	RCTs	_	7 days	Ranitidine	14.21 ± 5.44	25	35.1 ± 14.1	19/6	Ranitidine 50 mg, IV, every 6 hours
					Sucralfate	13.34 ± 6.03	25	27.5 ± 16.6	13/12	Sucralfate 1 g, every 6 hours
Kantorova et al. [23], 2004	Czech Republic	RCTs	Feb. 2000-June 2002	Until discharge or death	Famotidine	19.1 ± 9.3	71	47.0 ± 17	44/27	Famotidine 40 mg, IV, every 12 hours
					Sucralfate	18.8 ± 8.1	69	51.0 ± 18	50/19	Sucralfate 1 g, every 6 hours
Darlong et al. [24], 2003	India	RCTs	_	_	Ranitidine	_	24	44.0+18.5	11/13	Ranitidine 50 mg, IV, every 8 hours
					Sucralfate		21	39.5+15.2	14/7	Sucralfate 1 g, every 8 hours
Cook et al. [16], 1998	Canada	Multicenter RCTs	Oct. 1992-May 1996	Until discharge or death	Ranitidine	24.7 ± 7.1	596	58.8 ± 18.1	369/227	Ranitidine 50 mg, IV, every 8 hours
					Sucralfate	24.6 ± 7.3	604	58.7 ± 18.7	354/250	Sucralfate 1 g, every 6 hours
Thomason et al. [25], 1996	USA	RCTs	Nov. 1990-May 1994	27.3 days	Ranitidine	17.0	136	31.0	_	Ranitidine 150 mg/day, continuous IV
					Sucralfate	19.0	140	27.7	_	Sucralfate 1 g, every 6 hours
Prod'hom et al. [26], 1994	Switzerland	RCTs	Jan. 1989-Jan. 1991	_	Ranitidine	16.8 ± 8.6	80	52.2 ± 18.1	54/26	Ranitidine 150 mg/day, continuous IV
				_	Sucralfate	17.2 ± 8.6	83	46.4 ± 17.5	56/27	Sucralfate 1 g, every 4 hours
Pickworth et al. [27], 1993	USA	RCTs	Jan. 1989-Aug. 1991	Until discharge or death	Ranitidine	18.1 ± 6.5	44	27.3	60/23	Ranitidine 50 mg, IV, every 6 hours
					Sucralfate		39	26.8		Sucralfate 1 g, every 6 hours
Ruiz-Santana et al. [28], 1991	Spain	RCTs	Dec. 1998-Jan. 1990	Until discharge or death	Ranitidine	15 ± 5	19	39.0 ± 17	14/5	Ranitidine 50 mg, IV, every 6 hours
					Sucralfate		24	37.0 ± 18	20/4	Sucralfate 1 g, every 4 hours
Eddleston et al. [29], 1991	UK	RCTs	_	_	Ranitidine	12.4 ± 1.5	30	54.1 ± 3.1	17/13	Ranitidine 50 mg, IV, every 6 hours
			_	_	Sucralfate	11.6 ± 1.3	30	44.3 ± 3.5	21/9	Sucralfate 1 g, every 6 hours
Laggner et al. [30], 1989	Austria	RCTs	_	Until discharge or death	Ranitidine	_	16	60 ± 15	7/9	Ranitidine 50 mg, IV, every 4 hours
			_		Sucralfate	_	16	47 ± 19	11/5	Sucralfate 1 g, every 4 hours^a^

The range of Acute Physiology and Chronic Health Evaluation II (APACHE II) scores was 0 to 71, with higher scores indicating a more severe

illness. ^a^If pH was less than 3.5, ranitidine 100 mg. IV, intravenous (injection); RCT, randomized controlled trial.

### Quality assessment of the trials

Treatment assignments were the typical method of 'randomization' across studies in this meta-analysis. Randomized treatment allocation sequences were generated in six trials [16,22,23,25-27]; for the other four trials [24,28-30], the method reported was judged to be unclear on the basis of the available documents. The original papers clearly stated that blinding was conducted across the studies and therefore the outcome measurements were not likely to be influenced by lack of blinding. The numbers and reasons for withdrawal/dropout were reported in detail across trials. None of the trials had extreme imbalances at baseline or was stopped early. Thus, the trials were free of other sources of bias. Therefore, six studies [16,22,23,25-27] were categorized as low risk of bias (plausible bias unlikely to seriously alter the results), and the other four studies [24,28-30] were categorized as unclear risk of bias (plausible bias that raises some doubt about the results). An overview of the quality appraisal is shown in Table 2.

**Table 2:** Quality assessment of studies included in the meta-analysis

Study	Adequate sequence generation	Allocation concealment	Blinding	Incomplete outcome data addressed	Selective outcome reporting	Free of other bias	Summary risk of bias
Prakash et al. [22], 2008	Yes	Yes	Yes	Yes	Yes	Yes	Low
Kantorova et al. [23], 2004	Yes	Yes	Yes	Yes	Yes	Yes	Low
Darlong et al. [24], 2003	Yes	Unclear	Unclear	Yes	Yes	Unclear	Unclear
Cook et al. [16], 1998	Yes	Yes	Yes	Yes	Yes	Yes	Low
Thomason et al. [25], 1996	Yes	Yes	Yes	Yes	Yes	Yes	Low
Prod'hom et al. [26], 1994	Yes	Yes	Yes	Yes	Yes	Yes	Low
Pickworth et al. [27], 1993	Yes	Yes	Yes	Yes	Yes	Yes	Low
Ruiz-Santana et al. [28], 1991	Yes	Unclear	Yes	Yes	Yes	Unclear	Unclear
Eddleston et al. [29], 1991	Yes	Unclear	Yes	Yes	Yes	Unclear	Unclear
Laggner et al. [30], 1989	Yes	Unclear	Yes	Yes	Yes	Unclear	Unclear

### Overt bleeding

**Figure 2:**
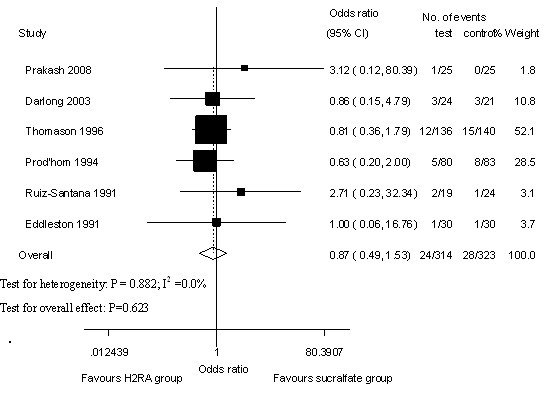
**Overt bleeding of histamine-2-receptor antagonists (H_2_RA) versus sucralfate**. Fixed-effects model of odds ratio (95% confidence interval, or CI) of overt bleeding associated with H_2_RA compared with sucralfate is shown.

Six RCTs [22,24-26,28,29] compared the incidence of overt bleeding with H_2_RA and sucralfate. A total of 24 overt-bleeding events occurred among 314 patients (7.6%) in the H_2_RA group compared with 28 events among 323 patients (8.7%) in the sucralfate group. Compared with sucralfate therapy, H_2_RA therapy was not associated with a significant reduction in the risk of overt bleeding, and no heterogeneity was detected across trials (OR 0.87, 95% CI 0.49 to 1.53, *P *= 0.623, *P *of heterogeneity = 0.882, I^2 ^= 0.0%; Figure 2).

### Clinically important gastrointestinal bleeding

The events of clinically important GI bleeding were explored in three studies [16,23,30]. The pooled analysis of the clinically important GI-bleeding rate for H_2_RA versus sucralfate showed a significant heterogeneity (*P *= 0.074, I^2 ^= 61.7%). On the basis of the results of the sensitivity analysis, one study [30] was excluded. The subsequent analysis was based on two trials [16,23], and a total of 12 clinically important GI-bleeding events occurred among 667 patients (1.8%) in the H_2_RA group compared with 26 events among 673 patients (3.9%) in the sucralfate group. Nevertheless, the sample sizes were highly variable across trials, one of which contained more than nine times as many subjects as the others. Therefore, it was inappropriate to pool the data.

### Ventilator-associated pneumonia

**Figure 3:**
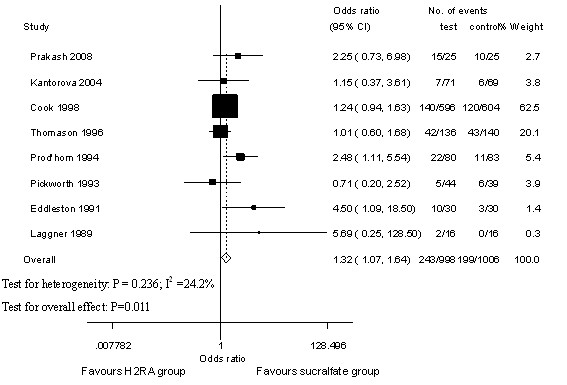
**Ventilator-associated pneumonia of histamine-2-receptor antagonists (H_2_RA) versus sucralfate**. Fixed-effects model of odds ratio (95% confidence interval, or CI) of ventilator-associated pneumonia associated with H_2_RA compared with sucralfate is shown.

VAP data required for meta-analysis was available from eight studies [16,22,23,25-27,29,30]. The incidence of VAP in the H_2_RA group was 243/998 (24.4%) and that of the sucralfate group was 199/1,006 (19.8%). Pooled analysis of OR showed that VAP was significantly less prominent among participants receiving sucralfate in relation to H_2_RA, and the results were robust and there was no evidence of heterogeneity (OR 1.32, 95% CI 1.07 to 1.64, *P *= 0.011, *P *of heterogeneity = 0.236, I^2 ^= 24.2%; Figure 3).

### Subgroup analyses

Three trials [22,25,26] that included a total of 373 patients (H_2_RA, *n *= 185; sucralfate, *n *= 188) provided data to allow us to conduct subgroup analyses based on early- or late-onset pneumonia. A total of 28 early-onset pneumonia events occurred among 185 patients (15.1%) receiving H_2_RA therapy compared with 41 events among 188 patients (21.8%) receiving sucralfate therapy, and we found no significant difference according to the incidence rates of early-onset pneumonia (OR 0.62, 95% CI 0.36 to 1.07, *P *= 0.085). Only for the outcome of late-onset pneumonia did we find a significant difference suggesting higher frequencies of late-onset pneumonia with the patients receiving H_2_RA compared with those receiving sucralfate (H_2_RA: 36/185 [19.5%]; sucralfate: 10/188 [5.3%]; OR 4.36, 95% CI 2.09 to 9.09, *P *< 0.001). No heterogeneity was detected in those two subgroup analyses, and *P *values were 0.362 and 0.725, respectively.

### Gastric colonization

Four studies assessing 413 participants who were randomly assigned to receive H_2_RA therapy (*n *= 206) or sucralfate therapy (*n *= 207) provided the information on gastric colonization [22,23,26,29]. Pooled analysis of OR showed that there was a significant difference of gastric colonization between the two groups (OR 2.72, 95% CI 1.80 to 4.13), with heterogeneity among the trials (*P *of heterogeneity was less than 0.001). Sensitivity analysis indicated that the outcome was not robust until we excluded the study by Prakash and colleagues [22], and so the source of heterogeneity could be mainly from that trial. The heterogeneity disappeared after the removal of that study, and the remaining trails showed that there was a significant difference in gastric colonization between H_2_RA and sucralfate (OR 2.03, 95% CI 1.29 to 3.19, *P *= 0.002, *P *of heterogeneity = 0.298, I^2 ^= 17.5%).

### Intensive care unit mortality

**Figure 4:**
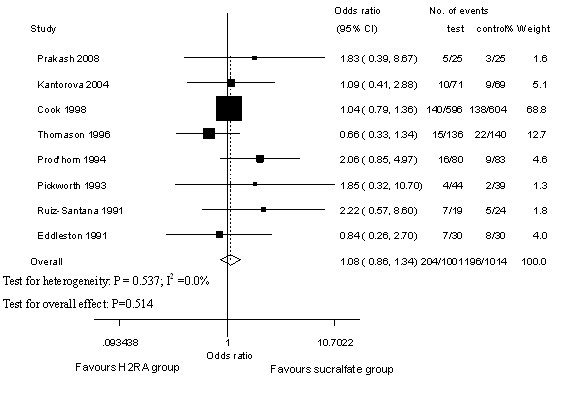
**Intensive care unit mortality of histamine-2-receptor antagonists (H_2_RA) versus sucralfate**. Fixed-effects model of odds ratio (95% confidence interval, or CI) of intensive care unit mortality associated with H_2_RA compared with sucralfate is shown.

The ICU mortality rate during the active study period for participants who were treated with H_2_RA was 204/1,001 and that of participants treated with sucralfate was 196/1,014, according to eight trials with available data [16,22,23,25-29]. Compared with the OR of mortality associated with sucralfate, the OR of mortality associated with H_2_RA was 1.08 (95% CI 0.86 to 1.34, *P *= 0.514), indicating that the result was not statistically significant comparing H_2_RA with sucralfate in reducing overall ICU mortality. No heterogeneity across trials was detected by the χ^2 ^test, and the *P *value was 0.537 (I^2 ^= 0.0%; Figure 4).

### Publication bias

**Figure 5:**
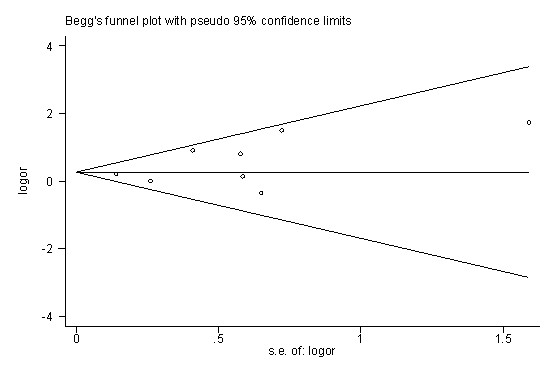
**Publication bias of the meta-analysis**. Publication bias for the outcome of ventilator-associated pneumonia in studies of the effects of histamine-2-receptor antagonists versus sucralfate on stress ulcer prophylaxis in mechanically ventilated patients is shown. s.e., standard error.

Inspection of funnel plots and statistical tests for publication bias did not show an obvious effect of publication bias (Egger test, *P *= 0.208; Begg test, *P *= 0.536; Figure 5).

## Discussion

The potential differences among the prophylactic regimens of critically ill patients have evoked great interest from clinicians, scientists, and the public. During the past few decades, studies and overviews have investigated this topic, but consistent results have not been reported and no individual study has definitively established whether these agents decrease clinically important GI bleeding. The meta-analysis [13] suggested that ranitidine and sucralfate do not prevent GI bleeding in ICU patients. In contrast, the overview of Pérez and Dellinger [31] in 2001 still strongly recommended stress ulcer prophylaxis, particularly in patients with mechanical ventilation, hypotension, and coagulopathy. Moreover, although recognizing the duration of intubation was an important risk factor for the development of VAP, no meta-analysis comparing stress ulcer prophylaxis had analyzed when the pneumonic episodes had developed in the study participants. It was extremely important that VAP developing early or late after intubation might differ in the bacterial species that were recovered from the trachea [32-34] and that therefore were likely to be related to different pathophysiologic mechanisms.

We therefore included recently published studies and generated a meta-analysis to elucidate and quantitatively assess the differences in the effect of H_2_RA versus sucralfate on stress ulcer prophylaxis in mechanically ventilated patients in the ICU. The present study, in which we identified and evaluated 10 relevant RCTs comparing H_2_RA therapy versus sucralfate therapy, was based on individual patient data from 2,092 patients enrolled in RCTs conducted by independent investigators. Results of this meta-analysis demonstrated that, for patients with mechanical ventilation, a comparable incidence of overt bleeding was associated with H_2_RA in comparison with sucralfate. From our analysis, with all available articles, we confirmed the finding of single trials that sucralfate was associated with significantly lower rates of incidence of gastric colonization, VAP, and late-onset pneumonia relative to H_2_RA. The analysis demonstrated that equivalent incidence rates were observed between the two groups with regard to early-onset pneumonia and ICU mortality rate.

Results concerning gastric-bleeding prevention were consistent with the prior meta-analysis [12] and were replicated in the current analysis as we found no evidence that H_2_RA and sucralfate differ with respect to the prevention of overt bleeding. First, the reason for replication was that the two included trials of greatest weight also fulfilled Cook and colleagues' criterion of overt bleeding [12]. Second, the analysis of data was based on similar definitions of overt bleeding. Third, both of them used the fixed-effects model for the analytic strategy.

The present study showed a marked reduction in clinically important GI bleeding with H_2_RA (1.8%) in relation to sucralfate (3.9%). Nevertheless, it was inappropriate to pool the data given that the sample sizes were highly variable across trials, one of which contained more than nine times as many subjects as the other. There were several discrepancies between our study and the previous studies. First, the analysis of different data was based on different definitions of clinically important GI bleeding. Second, the participants who developed clinically important GI bleeding were not homogeneous in the included trails of study, which did not fulfill the 'clinically important GI bleeding' criterion that the trials themselves established [12]. In our study, we rigorously abstracted data from original studies published online, but we did not modify the data. In addition, combined and included studies definitely met the criterion of 'clinically important GI bleeding' as defined above in the 'Data extraction' section. In the present study, a definitive conclusion that critically ill patients undergoing mechanical ventilation ought to receive prophylaxis with H_2_RA or sucralfate to prevent clinically important GI bleeding could not be established.

With respect to VAP, a recent meta-analysis [13] suggested that sucralfate was associated with decreased incidence rates of VAP in comparison with H_2_RA, whereas another study [12] found only a trend toward a decreased incidence of VAP when sucralfate was compared with H_2_RA, but the trend was not statistically significant. The finding of our meta-analysis indicated that incidence rates of VAP were significantly more prominent in the H_2_RA group than in the sucralfate group (OR 1.32, 95% CI 1.07 to 1.64). Although no statistically significant difference in the incidence rates of early-onset pneumonia was found between groups, patients on H_2_RA were associated with an increased incidence of late-onset pneumonia (OR 4.36, 95% CI 2.09 to 9.09). In addition, patients receiving H_2_RA had higher magnitudes of gastric colonization than did patients receiving sucralfate (OR 2.03, 95% CI 1.29 to 3.19). Importantly, in spite of evidence of heterogeneity among trials, the heterogeneity would be expected as a result of chance; this was not surprising given the certain differences in target populations and methods. We postulated that the lower incidence of late-onset pneumonia in the sucralfate group appeared to be associated mainly with the fact that sucralfate did not alter the gastric pH, for the gastric pH has been shown to greatly affect the bacterial colonization of the stomach [35-37]. Thus, patients receiving this drug were able to maintain a low gastric pH and suppress bacterial growth. In that case, in early-onset pneumonia that developed during the first few days after intubation, the spectrum of bacteria (which mostly included oropharyngeal species) were probably considered to have been introduced in the trachea before or at the time of intubation.

All available trials were aggregated to evaluate the effect of H_2_RA and sucralfate on ICU mortality. In studies evaluating mortality, we observed similar rates of ICU mortality among patients receiving H_2_RA (204 of 1,001 [20.4%]) and those receiving sucralfate (196 of 1,014 [19.3%]), and there was no significant difference between groups (OR 1.08, 95% CI 0.86 to 1.34). Other investigators reported that development of VAP might lead to an additional 13 days in the ICU [38]. Although the frequencies of VAP were less prominent among participants receiving sucralfate in relation to H_2_RA, the effect of this type of pneumonia appeared to have no direct relation with mortality. More high-quality RCTs were needed to explore the associated factors of mortality in the ICUs.

To our knowledge, our study was the first attempt to summarize the available data on the comparison of H_2_RA and sucralfate effects on stress ulcer prophylaxis in mechanically ventilated patients in the ICU. There were several novel aspects in our study: early- and late-onset pneumonias were first evaluated through subgroup analysis, allowing the combination of comparable estimates. Furthermore, an advantage of our analysis was that the definitions of outcome measures were clearly defined in the present study and this resulted in precise results.

However, we do acknowledge that there are several limitations of the present study. First, the geographic regions covered in this meta-analysis include North America (the US and Canada) [16,25,27], Europe (the Czech Republic, Switzerland, Austria, and Spain) [23,26,28,30], and Asia (India) [22-24]. Therefore, our results have limited generalizability to other regions (for example, Africa and Latin America). Second, a small number of studies and participants in particular outcome measures were available. Although lower frequencies of clinically important GI bleeding were noted in the H_2_RA group (12/667 [1.8%]) compared with the sucralfate group (26/673 [3.9%]), the result would have been attributed to the definitive RCT published by Cook and colleagues [16] if the data of the two trials had been synthesized, and this probably limited the detection of the effect estimate. Therefore, it was inappropriate to pool the data. Finally, differences in APACHE II score and intervention dosage might have affected the outcome of patients' response to medical management and might have produced possible clinical heterogeneity.

## Conclusions

This meta-analysis demonstrated that, compared with sucralfate for the prevention of stress ulcer in mechanically ventilated patients, H_2_RA showed no differential effectiveness in treating overt bleeding but had the disadvantages of higher gastric colonization and VAP rates. In clinical practice, the increased risks of adverse effect had to be balanced against the benefits of treatment with H_2_RA while taking into account each patient's clinical circumstances. Larger prospective RCTs and additional African and Latin American studies of H_2_RA and sucralfate are warranted among patients with mechanical ventilation in order to allow firm conclusions to be drawn about clinical benefit and risks, particularly clinically important GI bleeding.

## Key messages

• The literature shows that histamine-2-receptor antagonists (H_2_RA) result in no differential effectiveness in treating overt bleeding but have higher rates of gastric colonization and ventilator-associated pneumonia on stress ulcer prophylaxis in mechanically ventilated patients in the intensive care unit.

• There is a lack of consensus in the literature in regard to the effect of H_2_RA versus sucralfate in treating clinically important gastrointestinal bleeding.

• Larger prospective randomized controlled trials and additional African and Latin American studies of H_2_RA and sucralfate are warranted and may have a positive impact on overall estimates.

## Abbreviations

APACHE II: Acute Physiology and Chronic Health Evaluation II; CENTRAL: Cochrane Central Register of Controlled Trials; CI: confidence interval; GI: gastrointestinal; H_2_RA: histamine-2-receptor antagonists; ICU: intensive care unit; OR: odds ratio; RCT: randomized controlled trial; VAP: ventilator-associated pneumonia.

## Competing interests

The authors declare that they have no competing interests.

## Authors' contributions

FG and YC conceived the study and helped with manuscript revisions. CL designed and performed searches. LW participated in the extraction and analysis of the data. JH was involved in drafting the manuscript and worked on manuscript revisions. All authors read and approved the final manuscript.
